# Supplementary material for: A results to action framework for community verification: A case study from a performance based financing program in Zimbabwe
Source: PLOS Glob Public Health. 2025 Aug 26;5(8):e0004027. doi: 10.1371/journal.pgph.0004027 (PMC12380330; doi:10.1371/journal.pgph.0004027)
Supplement: Attachment — Submitted filename: Response to reviewers_20241111.docx [file pgph.0004027.s004.docx]

# Response to reviewers - PLOS Global Public Health

## Reviewers #1

| **N** | **In Section:** | **Review** | **Response** | **Edited Text** |
| --- | --- | --- | --- | --- |
| 1 | Overall | It is unclear if this verification is for the national program and across all sites and regions or specific to a location or area or just Gates supported VMMC sites? Recommend specifying. | Thank you for this feedback – indeed this is unclear in the original. This study was not for the national program, but instead a grant to PSI that covered a portion of sites. We’ve further clarified in the “PBF Program Design” section. | Starting in 2015, the Bill & Melinda Gates Foundation supported Zimbabwe’s VMMC program through a series of grants to Population Services International (PSI), in partnership with the MoHCC. |
| 2 | Overall | Recommend updating ‘community verification’ to ‘patient verification’ because this introduces confusion to the reader because the verification is happening at the individual level and not the community which implies another type of verification. This remains unclear beyond the abstract with references to community verifiers and also references to verification of patients. | We completely agree that ‘patient verification’ may be clearer to some readers, but we thought it would be best to remain consistent with the PBF literature, which use this term; kindly see the manuscripts below. We improved the text in a few ways to clarify on first mention, both in the abstract and the body, for further review by the reviewers.  11.Antony M, Bertone MP, Barthes O. Exploring implementation practices in results-based financing: the case of the verification in Benin. BMC Health Serv Res. 2017 Mar 14;17(1):204.  21.Turcotte-Tremblay AM, Gali-Gali IA, De Allegri M, Ridde V. The unintended consequences of community verifications for performance-based financing in Burkina Faso. Soc Sci Med. 2017 Oct 1;191:226–36.  31.Zitti T, Ridde V, COULIBALY A. From Measuring User Satisfaction to Improving Quality of Care: Community Verifications and Performance-Based Financing in District Hospitals in Mali. 2022. | Background: Performance-based financing (PBF) is a funding strategy that pays for outcomes rather than the cost of inputs. Verification through facility records (quantity verification) and patient interviews in communities (community verification) is a known cornerstone of PBF to ensure reported results are accurate.  …  Typically, PBF verifications include reviews of paper-based facility records (also called quantity verification) to ensure records are complete and interviews with patients in communities (called community verification, client tracing) to ensure records represent delivered services since records could be fabricated (10,11).  …  Community verification: Among facility records that were sufficiently complete, 10% were randomly selected for patient interviews in communities (2.5% of reported VMMCs). |
| 3 | Abstract | Recommend specifying which records are being verified with the patient records (i.e. MOH, national, regional, etc.) | Please see our updates to clarify in the first sentence after “Quantity verification:” | Quantity verification: Verifiers visited health facilities to quantify the alignment between the number of sufficiently complete facility records and the aggregated totals per month and facility that were stored in PSI’s health management information system (DHIS2). |
| 4 | Abstract | “the paths and processes that led to the results” – this could be removed since you don’t explain the “paths and processes” in the rest of the abstract. | Thank you, yes. Done |  |
| 5 | Abstract | “For a VMMC program in Zimbabwe that took place from 2016-2018, we performed a mixed method verification including quantity verification, community verification, and verifier interviews to help understand the findings.” Recommend updating to improve clarity: “We performed a mixed method verification including quantity verification, community verification and interviews to verify records for the national (?) VMMC program from 2016 – 2018.” | Done. We adjusted to clarify. | Methods: We performed a mixed method verification including quantity verification and community verification to verify reported results for Population Services International’s VMMC program in Zimbabwe from 2016 – 2018. We also interviewed verifiers to help understand the findings and we assessed the correlation between quantity and community verification performance scores at the facility-level to see whether facilities that have strong record keeping tended to also have strong validation from patients and vice versa. |
| 6 | Abstract | For the sentence: “We also assessed the correlation between the quantity and community verification performance scores to see whether facilities that have strong record keeping tended to also have strong validation from patients and vice versa”, recommend either removing this reference to performance scores since it isn’t clear how these scores were calculated in the abstract. | We added detail that the scores are based on the two main activities, for your review. | Methods: We performed a mixed method verification including quantity verification and community verification to verify reported results for Population Services International’s VMMC program in Zimbabwe from 2016 – 2018. We also interviewed verifiers to help understand the findings and we assessed the correlation between quantity and community verification performance scores at the facility-level to see whether facilities that have strong record keeping tended to also have strong validation from patients and vice versa. |
| 7 | Abstract | Information is needed on how records were selected for this data verification exercise | We have added detail about the sampling strategy. We didn’t go into detail given it’s a purposeful sample, but do let us know if more detail would be best. | A purposeful sample of records were selected to fulfill requirements from the funder, which was that 25% of records needed to be verified. At each of the four time points, PSI staff selected between 6 to 9 months of reported VMMCs, based on what was needed to be on track for the 25% requirement. |
| 8 | Abstract | “Of the 36,877 reported VMMCs, 94% were verified with facility records.” Were these nationally reported? If yes, please add to this sentence. | Yes, we clarified that the sample originated from DHIS2. | Results: Among the 36,877 VMMCs selected from DHIS2 for quantity verification, 94% of records were sufficiently complete.  …  Quantity verification: Verifiers visited health facilities to quantify the alignment between the number of sufficiently complete facility records and the aggregated totals per month and facility that were stored in PSI’s health management information system (DHIS2). |
| 9 | Abstract | For the sentence: “Community verifiers reported that some patients admitted providing incorrect contact information to avoid follow-up and most verifiers suspected staff had fabricated data.” Do you have any findings to support that staff fabricated data? Recommend not reporting that they ‘suspected’ which is not objective and presenting any data identified during the review to support that there was fabricated data. | We thank the reviewer for this comment, as the authors also wrestled with this issue. The purpose of the verifier interviews was to learn more about the 45% of patients that could not be reached for an interview—a figure that was alarming to PSI leadership & the funder as well as other PBF leaders who were familiar with the initial results. Were these patients missing due to no fault of PSI, or could it be that a portion of the VMMCs were not performed? The latter is plausible given the financial incentive introduced by the program, and—if it was true—would have notable implications for both this program and PBFs more broadly. PSI had not planned financially for the type of audit that would be able to prove data had been fabricated. Our goal with limited financial resources was to learn from the verifiers given their experience in the field and because they had no incentive to report suspected data fabrication if they did not suspect it. Therefore, we designed a semi structured questionnaire and we objectively report on their responses. While the verifiers did not have hard evidence about data fabrication by staff, our belief is that these findings will be important for PBF designers in the future; if they believe it’s important to know the reasons for missing patients with more certainty, they may consider investing more resources for a more rigorous verification. |  |
| 10 | Abstract | There are references to programs paying based on facility records, but these findings related to PBF aren’t mentioned in the results. The Conclusions should only present on information presented in the results otherwise it is unclear how these conclusions were drawn. | In the Conclusion of the Abstract, the statement of “Programs that pay based on facility records alone risk overpaying for services and misreporting performance” refers to the quantity verification results presented in this study, which were not a good proxy for the community verification results. For this program, if the funder would have only based payment on the facility records, the funder would have overpaid. |  |
| 11 | Methods | Study Setting: recommend adding sexual transmission to the last sentence “reduces the risk of female-to-male sexual transmission of HIV” | Done | The VMMC program in Zimbabwe launched in 2009, following randomized trials that showed circumcising men reduces the risk of female-to-male sexual transmission of HIV by up to 60% (23–25). |
| 12 | PBF Program Design | This section describes both PBF as supporting scale-up of VMMC but in the second sentence it refers to the activity as a ‘study’. Recommend specifying if this was a grant to support a study on the use of PBF to improve uptake of VMMC. | We rephrased the sentence under review to remove the word ‘scale’ and clarify that the funder supported the program through a series of grants to PSI. | Starting in 2015, the Bill & Melinda Gates Foundation supported Zimbabwe’s VMMC program through a series of grants to Population Services International (PSI), in partnership with the MoHCC. |
| 13 | PBF Program Design | It should be specified if the PBF included the national program and both PEPFAR supported and MoHCC-supported sites or just Gates supported sites or a mix of all. | We have further clarified that this was a separate grant in the Program Design section. | Starting in 2015, the Bill & Melinda Gates Foundation supported Zimbabwe’s VMMC program through a series of grants to Population Services International (PSI), in partnership with the MoHCC. |
| 14 | PBF Program Design | The sentence: “This study focused on VMMCs performed as part of a grant from May 2016 to April 2019, whereby PSI as well as facility staff were remunerated for each VMMC.” Were facility staff remunerated in addition to their regular pay? This should be clarified. | Yes staff were given a base pay, which we have now clarified. | Starting in 2015, the Bill & Melinda Gates Foundation supported Zimbabwe’s VMMC program through a series of grants to Population Services International (PSI), in partnership with the MoHCC. This study focused on VMMCs performed from May 2016 to April 2019, whereby PSI as well as PSI’s facility staff were remunerated for each VMMC; for staff, the incentivized payments were in addition to an unconditional base pay. |
| 15 | Community verification | Please specify how many attempts were made to contact VMMC clients before you considered the record ‘unverified’? | Yes of course. Done. | Community verification: Among facility records that were sufficiently complete, 10% were randomly selected for patient interviews in communities (2.5% of reported VMMCs). If patients were unavailable after three attempts of visiting the community, they were marked as unavailable. |
| 16 | Community verification | Specify age of minors in Zimbabwe | Minors were under 16 years old, which we have now clarified. | Patients or their guardians (in the case of minors under 16 years) were interviewed on the phone if possible or at their homes; responses were captured on a mobile phone |
| 17 | Community verification | Was there a reason only Prepex device use was asked about and not ShangRing? Prepex use slowly decreased in Zimbabwe after the WHO report was released in 2016 indicating 2 doses of TTCV is required prior to prepex use. I believe Shangring was introduced in 2019 and likely overlapped with the period of interest. If this study was only conducted at Gates funded sites maybe Prepex use continued for longer? | ShangRing wasn’t used as part of these activities. Prepex was used minimally in 2017, and from then onwards it was only surgery under this grant agreement. | Patients or their guardians (in the case of minors under 16 years) were interviewed on the phone if possible or at their homes; responses were captured on a mobile phone. |
| 18 | Community verification | It is confusing why verifiers had to rank their level of certainty about the potential for data fabrication when objective and quantifiable indicators were captured that would better indicate data fabrication. | To build upon our response to reviewer comment #9, we agree that the quantifiable indicators from community verification were the most valid results presented in the paper. From that process, we know that nearly half of patients were unavailable for an interview. Given this high rate, we asked verifiers *for their opinions* as to why this was the case; verifiers had no intrinsic or extrinsic reason for hypothesizing one reason over another. We have made this clearer with the new edits. We asked verifiers to rank their certainty level to both prompt them to thoughtfully reflect on their experiences/opinions and so we could assess how strongly they felt about their opinions. Having the Likert rankings enabled us to quantify their opinions. We believe the opinions of these experienced verifiers can inform hypotheses about why nearly half the patients were “missing” for the community verification and publishing them is important for the PBF community in order to strengthen verification methods. | To learn more about why some patients whose records were selected for community verification could not be interviewed, verifiers who had performed the community verifications were recruited and subsequently interviewed between 01/09/2020 to 01/10/2020. Verifiers who performed less than 10 patient interviews and who only worked during the first verification were excluded given program changes and recall bias concerns. Interviews were conducted in English over Zoom using a standardized semi-structured questionnaire. Verifiers were asked to provide Likert scale rankings regarding how frequently they encountered certain scenarios. They also ranked their certainty level regarding whether patients or VMMC staff had fabricated data, where 0 meant they had no suspicion and 10 meant they were certain. Verifiers were also asked open-ended questions to explain their rankings. Responses were transcribed in real time into an electronic survey and audio recorded; the recording was used to complete the transcription following the interview. |
| 19 | Results | For the sentence: “For the quantity verification, ninety-four percent (34,713/36,877) of selected records were sufficiently complete (C).” recommend quantifying what it means that the records were sufficiently complete. | Done | 1) The following 10 fields needed to be filled: facility name, date of procedure, patient name, patient date of birth, patient address, patient phone number, whether HIV testing was offered, patient consent, provider name, provider signature |
| 20 | Results | The description of the reasons verifiers suspected data fabrication is helpful but the indicators used to measure these suspicions are not standardized across reviewers but instead rely on reviewers individual experiences and interactions at the site level. The interpretation and reliability of these findings is limited. It is even mentioned that the verifiers didn’t have hard evidence about fabrication. There are substantial quantifiable results that should be the primary focus of the paper but these suspicions about data falsification take away from the validity and credibility of the other findings. Recommend removing with a mention in the discussion or improving the description of how these ‘suspicions’ were captured uniformly across sites in the Methods. | We are grateful for your suggestion; we have now improved the description of how verifier suspicions were captured uniformly across sites in the Methods section. We did use a standardized tool to gather verifier’s suspicions with the Likert scale & certainty level scale.  As in all Likert scale questions that ask for respondents’ perspectives, the measures are standard but how responders interpret the measures they are reporting against may vary. This is one of the limitations of survey research using Likert scales. To mitigate this, we consistently asked verifiers to describe their experiences and explain their opinions to contextualize the quantitative response given by verifiers.  We appreciate the reviewer’s positive feedback on the substantial quantifiable results. The quantifiable results suggest that *nearly half* of the patient records chosen for verification could not be verified (via a patient interview), which could challenge the underlying rationale for PBF programs. The methods employed in this verification are very common in PBF programming; it is unheard of to perform the detective work that would be necessary to identify fraud with certainty. Our paper suggests that perhaps more discussions in this regard are worth having among PBF designers and funders. If there suspicions are accurate, it would mean large sums of funding were paid out in error. | Interviews were conducted in English over Zoom using a standardized semi-structured questionnaire. Verifiers were asked to provide Likert scale rankings regarding how frequently they encountered certain scenarios. They also ranked their certainty level regarding whether patients or VMMC staff had fabricated data, where 0 meant they had no suspicion and 10 meant they were certain. Verifiers were also asked open-ended questions to explain their rankings. |

## Reviewers #2

| **N** | **In Section:** | **Review** | **Response** | **Edited Text** |
| --- | --- | --- | --- | --- |
| 21 | Verification Design | The article looks nice, you may only need to improve on the study design, it's not clear on the designs you employed.   - You used MEASURE Evaluation verification tool, you need to indicate version and link to source. - You can also add what you customized on the tool. | Thank you for these requested improvements. We edited the text to clarify the version of the tool we used. After reflection, we also changed the language to clarify that we used a custom tool that was broadly based on MEASURE Evaluation’s tool. As described in the first sentence in “Quantity Verification”, we simply compared the number of sufficiently complete records (numerator) to the reported aggregated totals (denominator). MEASURE’s tool adds complexity by comparing the facility records to what was reported to the region first, and then to headquarters. But the funder decided to skip this step—as their priority was simply what was reported at the highest level. | Quantity verification: Verifiers visited health facilities to quantify the alignment between the number of sufficiently complete facility records and the aggregated totals per month and facility that were stored in PSI’s health management information system (DHIS2). They recorded the verification results on a custom paper form based broadly on the July 2009 version of MEASURE Evaluation’s Routine Data Quality Assessment tool (26) and then supervisors entered the results into Excel. |
|  |  | May you add a table or figure that shows correlation test. | The two sets of results are all that was computed for the correlation test. Do let us know if there are additional details we can add to help clarify. |  |
| 22 | Results | On qualitative results you need to add more information on how falsified information was handled. | The information reported in the results were community verifier suspicions that some of the VMMCs reported were fabricated. These results were shared through the manuscript collaboration process among researchers from the University of Washington and implementing partners (PSI & MOHCC). Reported verifiers’ perceptions of fabricated data were also shared via programmatic reports written by some of the authors during the implementation. Verifiers’ responses to survey responses were reported in aggregate; individual participant responses were de-identified. While we hope that PSI, MOHCC, Gates foundation and others working in the PBF space will use the results to strengthen their program, such actions were outside of the scope of this study. |  |
