# Supplementary material for: A results to action framework for community verification: A case study from a performance based financing program in Zimbabwe
Source: PLOS Glob Public Health. 2025 Aug 26;5(8):e0004027. doi: 10.1371/journal.pgph.0004027 (PMC12380330; doi:10.1371/journal.pgph.0004027)
Supplement: Attachment — Submitted filename: Response to reviewers_20250417.docx [file pgph.0004027.s005.docx]

# Response to reviewers - PLOS Global Public Health

## Reviewers #3

| **In Section:** | **Review** | **Response** | **Edited Text** |
| --- | --- | --- | --- |
| Title | Title needs to precisely reflect the issue. | We have proposed a new, more direct title, for your review. | A RESULTS TO ACTION FRAMEWORK FOR COMMUNITY VERIFICATION: A CASE STUDY FROM A PERFORMANCE BASED FINANCING PROGRAM IN ZIMBABWE |
| Abstract | It is better to use recent data.  It is till 2019 only, why not up 2024 data. | We agree but unfortunately, we were unable to use more recent data because the authors that are not from Zimbabwe were only involved in the specific grant described in the paper. Since the purpose of the paper is to explore verification processes/challenges with PBF programs (rather than coverage rates per se), we believe the content will still be useful to those using or considering PBF programs in spite of the 2019 data cut-off point. |  |
| Table 1 | See all table data and try to percentage and figure data consistent. | We have reviewed the table data and have consistently used percentages and numerical data where applicable/appropriate. We apologize but are not understanding how the reviewer is suggesting we improve the table. Kindly clarify and we will incorporate. |  |
|  | How is there an N of 3623 instead of 3676? | We have updated the text to clarify the reason for missing data for two variables. | *Note: In the data entered from the facility, 53 patients had missing data for the method. If these patients were interviewed, the method was automatically counted as meeting the funder’s requirements so as to not penalize for the missingness. 17 patients were from a facility where the base funding was unavailable. |

## Reviewers #4

| **In Section:** |  | **Response** | **Edited Text** |
| --- | --- | --- | --- |
| Title | I think you need to revise the title of the article, because it doesn't necessarily reflect what you're saying. In your article, the results of the community check were not used to take action, nor to punish possible fraud. | We have adjusted the title to focus on the framework and made edits to clarify how the quantitative results were used. Note that the following response from the previous review round was in regards to the *qualitative* results; we suspect there was a misunderstanding that this applied to the quantitative results.   | This study focused on VMMCs performed from May 2016 to April 2019, whereby PSI as well as PSI’s facility staff were remunerated for each VMMC; for staff, the incentivized payments were in addition to an unconditional base pay. Each VMMC patient was documented with one paper record at the facility. Initial payments to staff were made based on the number of paper records. If the quantity verification or community verification activities described below identified unvalidated VMMCs, then a staff's future payments were reduced based on a discount factor. If unvalidated VMMCs were deemed to be the result of fraud, the responsible staff were additionally dismissed. |
| Methods | Can you specify who did what in the article? Specify who made the collection tools, data processing and analysis, etc. | We have included a paragraph summarizing the methodological roles of authors. | Authors TG, TK and MM developed the data collection tools for quantity and community verification and oversaw data collection. TG, GO, and LE created the data collection tools for the verifier interviews. TG and LE collected, processed and led the analysis of the data. |
|  | In your case, this survey was not carried out. Why not? | We apologize but are not understanding – which survey are you referring to? Kindly clarify and we will incorporate. Two surveys were carried out – the community verification survey and a survey afterward with the verifiers who performed the community verification survey. We summarize in the article when a respondent was selected but not interviewed. |  |
| Methods | What about data saturation? | We are confident that we reached data saturation for the verifier interviews for two reasons. First, the majority of the selected verifiers were interviewed (34/37 invited); those we did not invite had either interviewed few patients or worked many years before. Second, the codes we created to capture the verifier’s responses did not change or increase after ~ 3/4 of the verifiers had been interviewed—meaning no new themes, insights, or patterns emerged in the last 1/4 of interviews. |  |
|  | What research design was used for the qualitative – phenomenological, grounded theory, case study, etc.? | Per John Creswell’s mixed method framework, the research study utilizes an explanatory sequential design in that the purpose is to use qualitative data from purposefully sampled individuals to explain certain aspects of the previously collected quantitative results. The qualitative data was analyzed with a thematic analysis approach. |  |
| Methods | Can you draw up a theoretical model of the community verification process? | While we agree that such a model would be a great contribution to performance-based financing projects, we propose that its best to not add a second framework to the paper to keep the focus on the actions to take based on community verification data—the primary goal of this effort to improve practice in this field. That said, we will keep this in mind for future publications. |  |
| Methods | How were the auditors chosen? On what basis were PSI and Ministry of Health auditors chosen? | Audit leads included 1) employees/leaders within PSI’s M&E department, a central team that played an internal oversight role, and 2) MOHCC VMMC representatives who were appointed to their position based on longstanding experience with VMMC. The leads both provided oversight but also performed the audit activities themselves.  The rest of the auditors were either more junior employees from PSI’s M&E department or contractors of PSI’s M&E department. Contractors were primarily hired for the community verification which required more staff resources than employees could handle. Most of the time those hired had routinely worked for PSI on past projects, so were well vetted for the work. |  |
| Methods | Can you include a table showing the number of verifiers per period and per subcontractor? | We have added a new table to clarify. | Among all verifiers for the community verification (Table 1), those who performed less than 10 patient interviews and who only worked during the first verification were excluded given program changes and recall bias concerns. Interviews were conducted in English over Zoom using a standardized semi-structured questionnaire. Verifiers were asked to provide Likert scale rankings regarding how frequently they encountered certain scenarios. They also ranked their certainty level regarding whether patients or VMMC staff had fabricated data, where 0 meant they had no suspicion and 10 meant they were certain. Verifiers were also asked open-ended questions to explain their rankings. Responses were transcribed in real time into an electronic survey and audio recorded; the recording was used to complete the transcription following the interview. Additional information regarding the ethical, cultural, and scientific considerations specific to inclusivity in global research is included in the Supporting Information. **Table 1. Verifiers for the community verification** **Verification Time Point** \| **Number of Verifiers**; 1 \| 23; 2 \| 40; 3 \| 31; 4 \| 28 |
| Methods | How were the subcontractors chosen? How were the auditors paid? | There were no subcontractors; verifiers were either employees or direct contractor hires of PSI. All verifiers were paid a daily rate for the time they spent in the field. | Initial payments were made based on the number of records and then adjustments (reductions or increases) were applied as necessary following the ex-post verification activities; PSI dismissed staff who were subsequently deemed to have committed fraud. Verifiers were paid a daily rate for their time |
| Discussion | In your key words you talk about unintended consequences, you also say that voluntary medical male circumcisions is a taboo subject, what are the consequences of community verification on patients who didn't want this to be known in their community. | We have updated the Discussion to address this point. Community verification should not result in a patient’s services being revealed to others unless the verification doesn’t have (or verifiers don’t follow) strict protocols for confidentiality. We think this is an important topic to discuss, but outside of scope of our study. | Three primary recommendations are proposed in this framework for future research and program implementation. First, it is important that PBF programs reduce payment when a patient’s response does not plausibly match the facility record. The theoretical foundation for PBF is that it helps solve the principal-agent problem through paying the agent contingent on verified outcomes—so that the principal pays only for what has been achieved (4,35,36). The entire purpose of the verification is therefore to measure achievements (10). If community verification results are not used, it weakens the power of the incentive (11); there is virtually no way to identify cheating based on records alone (30). When it is not possible to verify whether patients received services (e.g., due to logistical/financial constraints or patients that will not consent to follow-up for privacy/stigma reasons), PBF may not be optimal in that setting (10). |
| Limitations | There is a great risk that the auditors have defrauded the surveys declared by telephone or at home, you'll have to put this as the limit of your work since you haven't checked in your turn whether the people who are declared as surveyed by the auditors really have been. | Yes this is a great point. We have added this point to the limitations. | This study has several limitations. First, the selection of facilities for quantity verification was purposeful, and as a result, so were the patients selected for community verification. Most notably, facilities that performed fewer VMMCs were not included so the findings might not generalize to those areas. The low portion of patients that were interviewed is important to understand challenges with finding patients, and yet reduces the internal validity of findings from interviewed patients. When considering the data quality of the community verification, it is possible that the verifiers fabricated some surveys due to common pressures to perform. However, verifiers were paid sufficiently and for their time (not per survey), and were not pressured to complete many surveys, so had low incentives to do so. |
