# Supplementary material for: A results to action framework for community verification: A case study from a performance based financing program in Zimbabwe
Source: PLOS Glob Public Health. 2025 Aug 26;5(8):e0004027. doi: 10.1371/journal.pgph.0004027 (PMC12380330; doi:10.1371/journal.pgph.0004027)
Supplement: Attachment — Submitted filename: Response to reviewers_20250704.docx [file pgph.0004027.s006.docx]

# Response to reviewers - PLOS Global Public Health

## Reviewers #1

| **Review** | **Response** | **Edited Text** |
| --- | --- | --- |
| There are some grammatical errors and inconsistencies that should be addressed during the final edits of the manuscript:  “Pearson Correlation coefficients” should be “Pearson correlation coefficients” (this should not be capitalized) | We corrected this error. | Pearson correlation coefficients |
| Consistency:  *Recommend harmonizing formatting of percentages (e.g., “94% (34,713 of 36,877)”). | We corrected this inconsistency. | 94% (34,713/36,877) |
| *Remove inconsistencies in decimal places (e.g., 0.68 instead of .68). | We corrected these inconsistencies. | (r=-.034, p=0.68) and linear regression (β=-.018, p=0.69) |
| The denominator in some percentages is unclear or inconsistent. For example, the authors report 9.5% (191/2010), but the earlier sentence says 151 people reported not being circumcised implying the remaining sample is 1,859, not 2,010.  Suggestion: Use consistent denominators and be explicit:  “Among the 1,859 patients who reported being circumcised…”  The authors could consider a table footnote or brief line in the text explaining that 151 were excluded for reporting not being circumcised. | We have updated the text and table title to make the denominator more clear/consistent. | 9.5% of interviewed patients (191/2010) reported being circumcised but provided an implausible answer regarding the time period, district, or method, as shown in the grey cells of Table 4; Table 4 summarizes the responses from interviewed patients, excluding the 151 that reported not being circumcised. 114 patients reported the facility and method plausibly, but not the date; 58 patients reported the facility and date plausibly but not the method. To learn about these 58 patients, verifiers were asked about the survey question that asks patients about the method used in their procedure; nearly one quarter of verifiers thought that patients at times may not have understood the difference between the two methods even after verifiers provided additional explanations. Finally, it is also possible that some verifiers interviewed the wrong patients: when asked whether they completed a survey even when they were not certain that the respondent was the right person, most said never, while four verifiers said rarely or sometimes.  Table 4. Reported date, method, and facility from interviewed patients who reported being circumcised |
